# Clinical relevance of the TECTA c.6183G>T variant identified in a family with autosomal dominant hearing loss: a case report

**DOI:** 10.3325/cmj.2023.64.329

**Published:** 2023-10

**Authors:** Ivona Sansović, Ana-Maria Meašić, Ljubica Odak, Mijana Kero

**Affiliations:** 1 Department of Medical and Laboratory Genetics, Endocrinology and Diabetology, Children’s Hospital Zagreb, University of Zagreb, School of Medicine, Zagreb, Croatia; 2 Department of Pediatrics, University Hospital Center Split, Split, Croatia

## Abstract

Missense variants in the α-tectorin gene (*TECTA*) cause autosomal dominant (DFNA8/A12) non-syndromic hearing loss (ADNSHL) and account for a considerable number of ADNSHL cases. According to genotype-phenotype correlation studies, missense variants in the zona pellucida (ZP) domain of α-tectorin predominantly cause mid-frequency HL. Here, we report on clinical exome sequencing results in a large family with early-onset, sensorineural, moderate-to-severe mid-frequency HL. We identified one heterozygous c.6183G>T variant near the ZP domain of *TECTA* segregating in five family members. This variant was previously reported as a variant of uncertain significance in a family with ADNSHL. On the basis of specific segregation in the currently studied family and the general guidelines of the American College of Medical Genetics and Genomics, we argue that the TECTA c.6183G>T variant should be considered a likely pathogenic cause of ADNSHL. This report adds to the knowledge on the rare c.6183G>T missense variant, which affects the immediate vicinity of the ZP domain in *TECTA*. Our findings highlight the importance of clinical evaluation in patients with familial HL and of studying family segregation when assessing the pathogenicity of a variant.

Autosomal dominant pathogenic variants in the α-tectorin (*TECTA*) gene result in characteristic mid- or high-frequency hearing loss (HL) depending on their position. These variants also account for a considerable number of cases of autosomal dominant non-syndromic hearing loss (ADNSHL). According to genotype-phenotype correlation studies, missense pathogenic variants in the C-terminal zona pellucida (ZP) domain predominantly cause mid-frequency HL, while pathogenic variants in the zonadhesin-like domain cause high-frequency HL (1,2). However, the majority of almost 500 missense variants known in *TECTA* are of uncertain clinical significance and are challenging to decipher.

Here, we report on the results of clinical exome analysis in a large family with prelingual, mid-frequency moderate-to-severe HL. The results revealed a c.6183G>T (p.Arg2061Ser) missense variant near the ZP domain in *TECTA* in all five affected family members. This variant was previously reported in one family with mid-frequency severe HL as a variant of uncertain significance (VUS) (3). On the basis of the specific HL phenotype and the autosomal dominant segregation of the variant in the studied family (and the applied PP1_ Strong criterion), we argue that the *TECTA* c.6183G>T variant should be reclassified as a likely pathogenic cause of ADNSHL.

## Case REPORT

**Figure 1:**
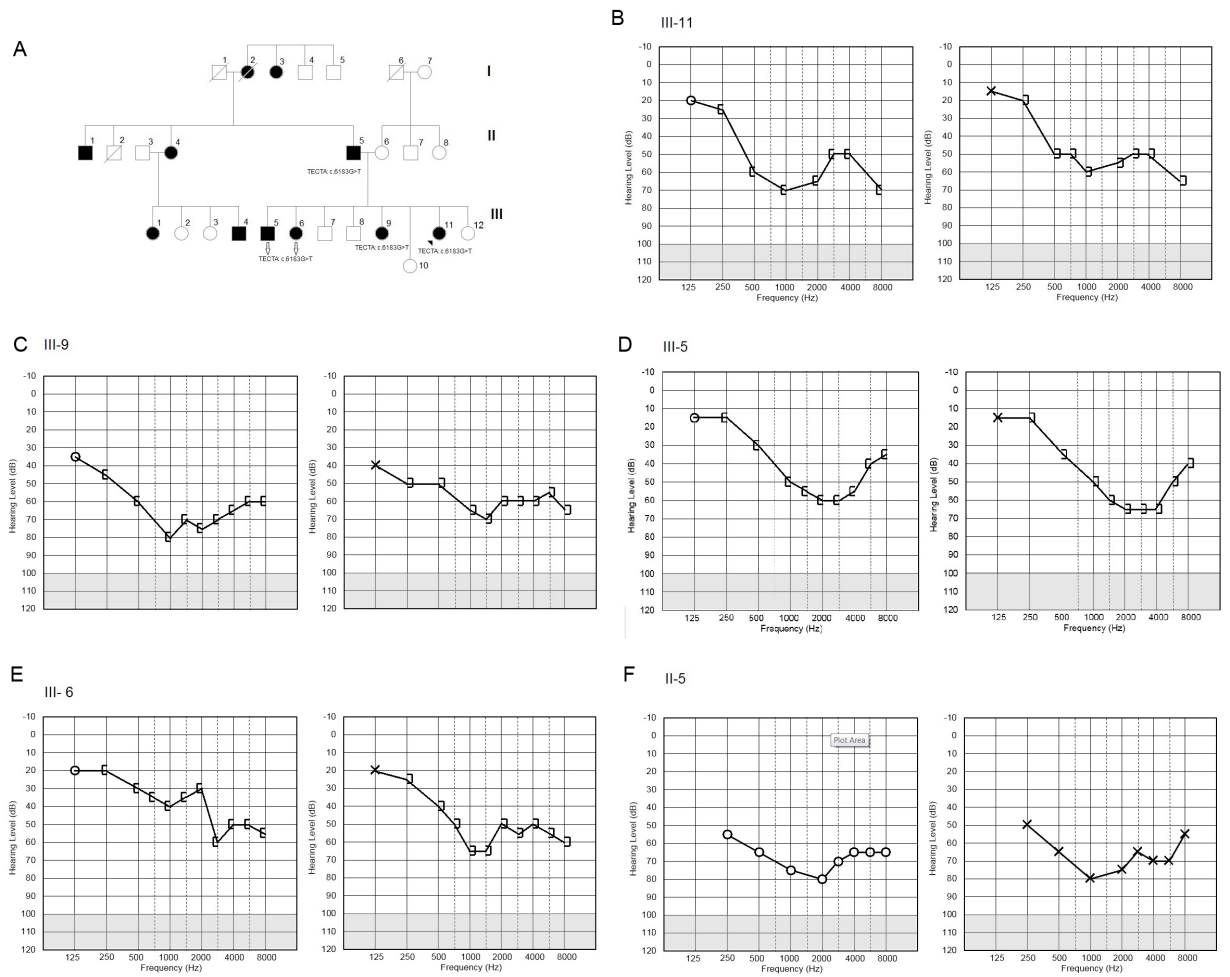
(**A**) The pedigree of a family with autosomal dominant hearing loss (HL) and the TECTA variant: III-11 is the proband (the arrow), II-5 is the proband’s father, III-5 is her brother, and III-6 and III-9 are her sisters. Black filled circles and squares indicate HL-affected family members. Family members genetically tested in this study were II-5, II-6, III-5, III-6, III-7, III-8, III-9, III-10, III-11, and III-12. Tonal audiometry for each family member with the *TECTA* variant (B-F). (**B**) audiogram of the proband at the age of 9 years (III-11; moderately severe HL); (**C**) audiogram of the first older sister at the age of 13 years (III-9; severe HL on right, and moderately severe on the left ear); (**D**) audiogram of the older brother at the age of 16.5 years (III-5; moderately severe HL); (**E**) audiogram of the second older sister at the age of 14 years (III-6; moderately severe HL); (**F**) audiogram of the father at the age of 44 years (II-5, severe HL). Circle: right ear hearing threshold; X-mark: left-ear hearing threshold; [-mark: right ear bone conduction masked; ]-mark: left ear bone conduction masked.

In September 2012, a 19-month-old girl was referred to the Department of Medical and Laboratory Genetics, Endocrinology, and Diabetology, Children’s Hospital Zagreb, due to familial congenital moderate bilateral sensorineural HL. She was one of eight children in her family, four of whom had HL. HL was also present in the father and six family members on the father's side. Five family members with HL (father and four children) underwent pure tone audiometry and speech evaluation at the Polyclinic for the Rehabilitation of Listening and Speech (SUVAG) in Zagreb. The phenotype was assessed at the Department of Medical and Laboratory Genetics, Endocrinology, and Diabetology, Children’s Hospital Zagreb on the basis of physical examinations and medical and family history, after which the medical geneticist referred the participants for genetic testing. A three-generation pedigree comprised 26 individuals, 10 of whom were genetically tested (siblings and the parents of the proband) (Figure 1A). Adult participants gave written informed consent for themselves and their children.

Sanger sequencing for clinically relevant variants in the coding region of the *GJB2* gene was performed only in the proband, while all other family members were tested with clinical exome sequencing (CES) as the first step in genetic evaluation. A CES library was generated with enrichment oligos by using Illumina DNA Prep with Enrichment (Illumina, San Diego, CA, USA) focusing on the exons of the 4813 genes associated with the disease (TruSight One Pane, Illumina).

ClinVar, LOVD, HGMD, and Hereditary Hearing Loss Homepage databases were searched for known clinically relevant variants (4-7). Variant annotation and analysis were performed with *in silico* prediction tools from the VarSome and Ensembl Variant Effect Predictor. All variants were classified according to the American College of Medical Genetics and Genomics/Association for Molecular Pathology (ACMG/AMP) guidelines as follows: pathogenic, likely pathogenic, VUS, likely benign, or benign (8).

In the exon 22 of the *TECTA* gene, a heterozygous missense variant NM_005422.2: c.6183G>T (p.Arg2061Ser) was identified in the proband (III-11) and other four family members with mostly stable, moderately severe-severe bilateral, sensorineural, mid-frequency prelingual HL: father (II-5), brother (III-5) and two sisters (III-6; III-9). This variant was not detected in the remaining five family members who did not have HL (Figure 1). All the affected participants wore bilateral hearing aids from an early age.

**Figure 2:**
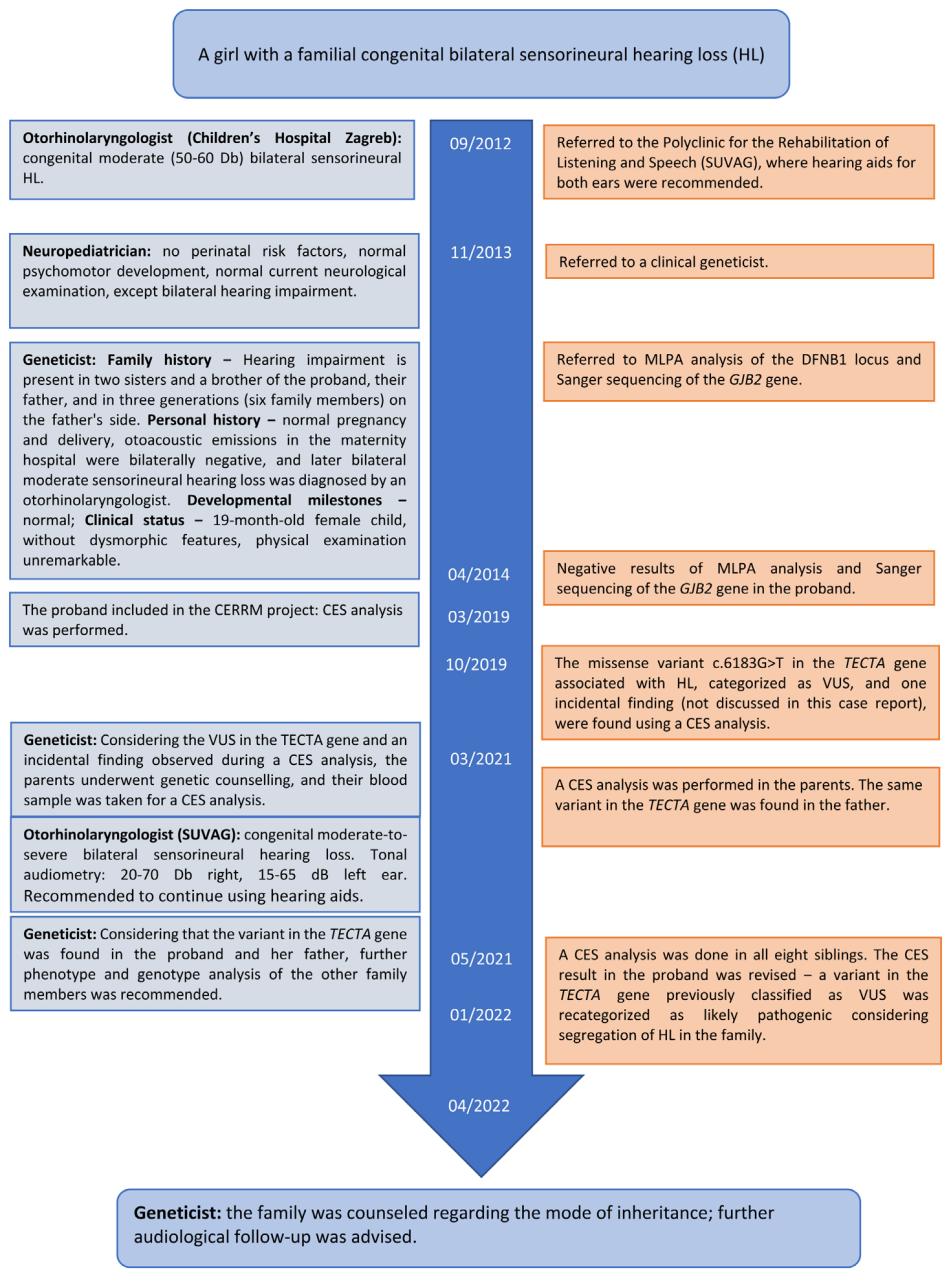
The timeline of relevant physical examinations, medical and family history, interventions and laboratory diagnostic assessment of the proband and her family. Abbreviations: CES - clinical exome sequencing; VUS - variant of uncertain significance; MLPA - multiplex-ligation dependent probe amplification; CERRM - Center of Excellence for Reproductive and Regenerative Medicine.

The clinical significance of the variant was assessed with the pathogenicity and conservation scores of c.6183G>T variant of the *TECTA* gene from VarSome and Ensembl Variant Effect Predictor databases. According to ACMG/AMP classification, this variant is likely pathogenic (PP1_ Strong, PP4, PM2, and PP3_ Supporting) (8). The substitution replaced conserved amino acid residue near the ZP domain of TECTA (Table 1). As this variant does not have gnomAD exomes and gnomAD genomes entry, it is classified as PM2_ supporting. According to the ACMG/AMP standard classification, PP3_ supporting rule was applied considering multiple pieces of computational evidence in favor of a damaging effect on the gene or its product (Table 1). Considering members of the families (but not the probands) from this and a previous study (3), segregation in five affected relatives for dominant inheritance was present, and PP1_ Strong rule can be applied (3,9). The timeline of diagnostic tests and interventions is shown in Figure 2.

**Table 1:** The *in silico* prediction pathogenicity and conservation scores for NM_005422.2 (TECTA): c.6183G>T variant*

Nucleotide change	Amino acid change	SIFT	Mutation taster	Mutation assessor	REVEL	LRT	PolyPhen	FATHMM	BLOSUM 62	CADD Phred hg19	GERP RS	phyloP100 way vertebrate
c.6183G>T	p.Arg2061Ser	D (0.555)	D (0.452)	M (0.638)	B (0.3019)	D (0.843)	D (0.966)	T (0.488)	-1	29.6	5.16	6.3

*Abbreviations: CADD – combined annotation-dependent depletion; BLOSUM62 – BLOcks SUbstitution Matrix; D – probably damaging or deleterious or disease causing; FATHMM – functional analysis through hidden Markov models; GERP – genomic evolutionary rate profiling, LRT – likelihood ratio test, M – medium; phyloP100way vertebrate – a conservation score based on multiple alignments of 100 vertebrate genomes; PolyPhen – polymorphism phenotyping; REVEL – rare exome variant ensemble learner; SIFT – sorting tolerant from intolerant; T – tolerated.

## Discussion

Similar to other individuals with missense variants in the ZP domain, participants with the c.6183G>T variant in this study also showed mostly stable, mid-frequency prelingual HL. The severity of HL ranged from moderate to severe. The proband’s father and one sister had severe HL. The same variant, classified as a VUS (PM2, PP3, and PP1_Supporting), was already described in a subject with severe mid-frequency HL at the age of 71 (3). The authors (3) suggested that *TECTA* pathogenic variants did not accelerate HL deterioration since the observed HL progression (0.3 dB/y) in patients with *TECTA* variants was similar to that in hearing controls, and probably reflected presbycusis (3). Therefore, we assume that the severity of HL in the father is also age-related.

Four affected family members in this pedigree and one affected family member from a previous study (3) (without probands) give a total of five affected members from two families, which indicates the status of PP1 strong evidence for the variant, ie, cosegregation with a disease in multiple affected family members in a gene definitively known to cause the disease (9).

The C-terminal ZP domain (residue T1805-N2059) is two amino acids away from the mutated R2061 residue detected in our study. A change from large-size and basic/positively charged arginine to small-size and polar/uncharged serine may affect the mechanical and electrical properties of the tectorial membrane. Although *TECTA*-mutant mice exhibit structural defects of the tectorial membrane, the molecular mechanism by which the pathogenic missense variants in the ZP domain cause mid-frequency HL is still unclear (10).

*TECTA*-associated ADNSHL is one of the most common ADNSHL subtypes (2). Considering 80% of *TECTA* variants of uncertain significance submitted to ClinVar database, it is difficult to prioritize missense *TECTA* variants causing AD-isolated HL. In the absence of functional studies that would elucidate the molecular mechanisms of *TECTA*-induced HL, when categorizing variants, one should take into account not only the results of *in silico* prediction tools, but also patients’ phenotypes and the study of family segregation. Yasukawa et al classified c.6183G>T *TECTA* variant as a VUS since the ACMG/AMP PP1 strong criterion could not be applied (3). Based on the specific HL phenotype and the autosomal dominant segregation of the variant in the studied family (and the applied PP1_ Strong criterion), we argue that the *TECTA* c.6183G>T variant should be reclassified as a likely pathogenic cause of ADNSHL.

In order to improve diagnostics and genetic information for the affected patients, it is necessary to resolve the molecular basis of the pathogenic missense variants, which are challenging to decipher. This study highlights the importance of clinical evaluation in patients with familial HL when assessing the pathogenicity of missense *TECTA* variants causing AD-isolated HL. This could contribute to a better genotype-phenotype correlation of rare missense variants in proximity to the ZP domain of the *TECTA* gene.
